# FAM175B promotes apoptosis by inhibiting ATF4 ubiquitination in esophageal squamous cell carcinoma

**DOI:** 10.1002/1878-0261.12474

**Published:** 2019-03-23

**Authors:** Yu Zhao, Yang Yu, Hengcun Li, Zheng Zhang, Shuilong Guo, Shengtao Zhu, Qingdong Guo, Peng Li, Li Min, Shutian Zhang

**Affiliations:** ^1^ Beijing Key Laboratory for Precancerous Lesion of Digestive Disease Department of Gastroenterology National Clinical Research Center for Digestive Disease Beijing Digestive Disease Center Beijing Friendship Hospital Capital Medical University Beijing China

**Keywords:** apoptosis, ATF4, ESCC, FAM175B

## Abstract

FAM175B is a reported regulator of p53 and suppresses tumorigenesis in numerous types of cancer, but very little is known about its function in esophageal squamous cell carcinomas (ESCCs), almost 70% of which exhibit mutations in p53. Here, we report that FAM175B expression is downregulated in high‐grade intraepithelial neoplasia (*t* = 2.44, *P* = 0.031) and ESCC (*t* = 5.664, *P* < 0.001) tissues relative to that in adjacent normal esophageal tissues. Exogenous expression of FAM175B in ESCC cells resulted in a decrease in proliferation rate, inhibition of colony formation, and an increase in apoptosis rate. Knockdown of FAM175B produced the opposite results. Furthermore, confocal microscopy and coimmunoprecipitation assay showed that Activating transcription factor 4 (ATF4) colocalized and interacted with FAM175B. Ubiquitination assays revealed that FAM175B inhibited ubiquitin‐dependent ATF4 degradation and elevated ATF4 protein level. Finally, luciferase reporter experiments further clarified that FAM175B promoted CHOP expression in an ATF4‐dependent manner. Accordingly, the proapoptotic activity of FAM175B was significantly rescued by treatment with si‐ATF4 and the CHOP inhibitor 4‐PBA. In summary, FAM175B inhibited ATF4 ubiquitination and promoted ESCC cell apoptosis in a p53‐independent manner. FAM175B expression loss may be an early diagnostic biomarker in ESCC patients.

**Abbreviations**
ATF4: Activating transcription factor 4
BRISC: BRCC36‐containing isopeptidase complex
CHX: cycloheximide
EAC: esophageal adenocarcinoma
EC: esophageal cancer
ERS: endoplasmic reticulum stress
ESCCs: esophageal squamous cell carcinomas
GEO: Gene Expression Omnibus
HGIEN: high‐grade intraepithelial neoplasia
PERK: protein kinase RNA‐like ER kinase

## 1 Introduction

Esophageal cancer (EC) is one of the most prevalent carcinomas worldwide, ranking sixth in cancer‐related mortality and eighth in newly diagnosed cases among all cancers (Jemal *et al*., 2011). Cancer incidence patterns reflect an increase in EC incidence, with 418 000 new cases diagnosed each year (Jemal *et al*., 2011). Esophageal adenocarcinoma (EAC) and esophageal squamous cell carcinoma (ESCC) are the two major subtypes of EC. ESCC has a higher incidence and mortality worldwide than EAC, especially in the Asian belt (Kamangar *et al*., 2006; Zhu *et al*., 2014). ESCC is a highly lethal disease, esophagectomy is indicated in only one‐third of patients at diagnosis, and nearly 85% of patients need continuous chemotherapy after surgery (Chadwick *et al*., 2016; Rodgers *et al*., 2007). However, therapeutic complications and operative mortality remain relatively high, and the quality of life may be significantly impaired. The average 5‐year survival rate of ESCC is only 35–45% (Thompson *et al*., 2008). Few approaches other than endoscopy are available for ESCC screening, resulting in a low detection rate and poor prognosis. Recently, biomarker discoveries have greatly improved the understanding and management of many types of cancer (Findlay *et al*., 2014). However, few biomarkers are available to facilitate early detection and treatment in ESCC.

The poor prognosis and low early detection rate of ESCC highlight the limitations in the study of EC biomarkers. The identification of biomarkers for the early detection of ESCC is urgently needed to predict recurrence, prognosis, and sensitivity to therapy (Min *et al*., 2017a,b). The ability to facilitate the early diagnosis and treatment of ESCC would help to guide endoscopic, surgical, and adjuvant treatment according to individual risk (Kamangar *et al*., 2006). Recent studies of ESCC biomarkers primarily focus on protein expression, copy number variation, loss of heterozygosity, DNA methylation, somatic DNA sequence, and single nucleotide polymorphisms (Findlay *et al*., 2014). EGFR, CCND1, and FGF3/INT2 have mainly been studied for the prediction of prognosis and treatment response (Findlay *et al*., 2014; Janmaat *et al*., 2006; Sunpaweravong *et al*., 2005; Suzuki *et al*., 1997; Takeshita *et al*., 2010) . Hypermethylation of CDKN2A and MGMT, which is associated with the loss of expression of these proteins and advanced histological grade of cancer, is relatively commonly reported in ESCC (Guo *et al*., 2006; Hibi *et al*., 2001; Kaz and Grady, 2014; Salam *et al*., 2009; Taghavi *et al*., 2010; Tokugawa *et al*., 2002; Xing *et al*., 1999; Zhang *et al*., 2003). However, ESCC biomarkers for early diagnosis are relatively rare, and some promising reported candidates greatly need robust validation (Zhao *et al*., 2017).

FAM175B, also called ABRO1/KIAA0157, is a member of the FAM175 family and was initially reported as a component of the BRCC36‐containing isopeptidase complex (BRISC) deubiquitinating enzyme complex. In this complex, FAM175B acts as a scaffold protein that can recruit proteins in the BRISC and promote its deubiquitinating activity (Cooper *et al*., 2010). In coronary artery disease, FAM175B expression was reported to be upregulated in the myocardial infarction area and to protect cardiac cells by reducing the number of Lys 63‐linked polyubiquitin chains on specific target proteins (Cilenti *et al*., 2011). Additionally, FAM175B was reported to specifically interact with three members of the activating protein‐1 (AP‐1) transcription factor family: JunD, Activating transcription factor 4 (ATF4), and ATF5 (Ambivero *et al*., 2012). ATF4 belongs to the ATF/cAMP‐responsive element‐binding protein transcription factor family and plays an important role in the unfolded protein response (UPR; Ameri and Harris, 2008; Harding *et al*., 2000). As a marked indication of endoplasmic reticulum stress (ERS), the UPR can promote the restoration of correct conformations from unfolded or misfolded proteins on one hand, but on the other hand, an excessive UPR can disrupt endoplasmic reticulum homeostasis to induce cell apoptosis (Laybutt *et al*., 2007). FAM175B was also reported to be a regulator of p53 in a cancer‐related study; FAM175B can suppress tumorigenesis by stabilizing p53 in a ubiquitin‐specific peptidase 7‐dependent manner (Zhang *et al*., 2014). However, to date, no study has revealed the p53‐independent carcinogenic activity of FAM175B in ESCC patients, among which almost 70% exhibit p53 mutation (Abedi‐Ardekani, 2014; Kawano *et al*., 2014; Makino *et al*., 2010). In this study, we evaluated the expression of FAM175B in ESCC and revealed its role as a tumor suppressor, which is mediated by inhibiting ATF4 ubiquitin‐dependent degradation and promoting cell apoptosis.

## 2 Materials and methods

### 2.1 Patients and tissue specimens

Esophageal squamous cell carcinomas tissues (HEso‐Squ150CS‐02) and paired adjacent noncancerous tissues were purchased from Outdo Biotech (Shanghai, China). An additional seven ESCC specimens and their paired adjacent high‐grade intraepithelial neoplasia (HGIEN) and noncancerous tissues were harvested after the surgical resection of patients who were clinically and histopathologically diagnosed with ESCC at Beijing Friendship Hospital, Capital Medical University. The experiments were undertaken with the understanding and written consent of each subject. The study protocol conformed to the standards set by the Declaration of Helsinki and was approved by the Ethics Committee of Beijing Friendship Hospital, Capital Medical University.

### 2.2 Immunohistochemistry

The FAM175B antibody for Immunohistochemistry (IHC) was purchased from Atlas Antibodies (Bromma, Sweden) (Cat No: HPA037591). After three deparaffinization steps for 15 min each in dimethylbenzene and a routine hydration step, the tissues were washed with PBS and then subjected to antigen retrieval. After treatment with 3% H_2_O_2_ for 10 min, the tissues were incubated in goat serum to block nonspecific binding and then in the primary antibody at 4 °C overnight. The next day, the tissues were incubated with the secondary antibody and were then washed and stained with 2,4‐diaminobutyric acid reagents. The staining extent was scored as follows: 0, negative; 1, 1–33%; 2, 34–66%; and 3, 67–100%. The staining intensity was scored as negative, low expression, medium expression, and high expression, which are denoted 0, 1, 2, and 3, respectively. Samples with an IHC score > 2 were identified as positive expression and ≤ 2 were considered negative. The correlation between FAM175B expression and clinical variables was evaluated. The IHC results were evaluated by pathologists, and the final scores were determined by multiplying the intensity score by the extent score.

### 2.3 Cell culture and transfection

Monoclonal cell lines KYSE30 and EC9706 (purchased from the Shanghai Institute of Cell Biology, Shanghai, China) were cultured in 40% RPMI 1640 medium, 40% Ham's F12‐K medium, and 20% FBS (Gibco, Waltham, MA, USA). Cells were maintained at 37 °C in a humidified atmosphere of 5% CO_2_, and transient transfection was performed using 5 μL of Lipofectamine 3000 with 2 μg of plasmids, 4 μL of P3000, or 5 μL of siRNA according to the manufacturer's protocol.

### 2.4 Plasmids, RNA interference, and antibodies

Human full‐length FAM175B cDNA was amplified by PCR and cloned into the pCMV‐HA vector (donated by the Academy of Military Medical Sciences). The siRNA sequence targeting FAM175B and the nonspecific siRNA sequence (purchased from GenePharma, Suzhou, China) were as follows: siFAM175B, 5′‐AUU CAC UAU UAG AAG GCU CUG‐3′; and siNC, 5′‐CUG GAC UUC CAG AAG AAC AUC‐3′. The following antibodies were used in this study: anti‐FAM175B mouse polyclonal antibody (ab68801; Abcam, Cambridge, MA, USA); anti‐FAM175B rabbit polyclonal antibody (HPA037591; Atlas Antibodies); anti‐ATF4 rabbit monoclonal antibody (D4B8, CST); anti‐ATF4 mouse monoclonal antibody (60035‐1‐Ig; Proteintech, Wuhan, China); anti‐ubiquitin (P4D1) mouse monoclonal antibody (#3936, CST); anti‐HA tag mouse monoclonal antibody (66006‐2‐Ig; Proteintech); anti‐CHOP rabbit polyclonal antibody (15204‐1‐AP; Proteintech); and anti‐GAPDH mouse monoclonal antibody (60004‐1‐Ig; Proteintech).

### 2.5 MTS and colony formation assays

For the MTS assay, cells were plated in 96‐well cell culture clusters (3000 cells per well) after transfection for 12 h. For quantitation of the cell proliferation rate, 20 μL of 5 mg·mL^−1^ MTS (thiazolyl blue) solution was added at 6, 24, 48, and 72 h after seeding, respectively, and incubated for 2 h at 37 °C, and then, the absorbance was measured at 490 nm in a microplate spectrophotometer. Data were normalized to the mean value of day 0, and proliferation curves were drawn. For the colony formation assay, cells were plated into 6‐well cell culture clusters (1000 cells per well) and incubated for 2 weeks in medium after transfection for 12 h. Then, the wells were washed with PBS and stained with hematoxylin. The colonies were photographed, and the number was counted using imagej software (Barcelona, Spain).

### 2.6 Apoptosis detection

Cells were washed with PBS after transfection for 48 h (knockdown treatment group and its negative control group for additional 500 μm H_2_O_2_ treatment for 24 h to induce apoptosis) and were then harvested using 0.25% trypsin (all supernatant liquid was collected). After centrifugation (1500 r.p.m.) for 5 min, the supernatant liquid was discarded, and the precipitation was resuspended in 1 mL of binding buffer for another centrifugation. Then, the cells were resuspended in 250 μL of binding buffer, and 5 μL each of annexin V‐FITC and 7‐AAD‐PerCP‐Cy5.5 (BD Biosciences, San Jose, CA, USA) were added. The cells were analyzed by a FACSVerse (Becton‐Dickinson, Franklin Lakes, NJ, USA) after incubation in the dark for 15 min.

### 2.7 Immunoprecipitation and western blotting

Cells for immunoprecipitation were transfected with pCMV‐FAM175B and lysed in lysis buffer (50 μm HEPES, 150 μm NaCl, 1 mm EDTA, 1% Triton X, and 10% glycerol) supplemented with protease inhibitor cocktail and were directly scraped from 75‐cm^2^ flasks using a brush. Cell lysates were then transferred to microcentrifuge tubes and incubated in an ice bath for 30 min followed by centrifugation at 4 °C and 9660 ***g*** for another 30 min. The lysates were Pre‐cleared with 20 μL of protein A/G agarose beads by rotating at 4 °C for 1 h. Then, the corresponding antibodies (an anti‐ATF4 rabbit monoclonal antibody for ATF4, an anti‐HA tag mouse monoclonal antibody for FAM175B, or IgG for the negative control) were mixed with the lysates and incubated on a rotator at 4 °C overnight followed by the addition of 30 μL of protein A/G agarose beads and rotation for 6 h at 4 °C. After the beads were washed with 500 μL of lysis buffer three times, SDS loading buffer without DTT was added, and proteins were denatured at 99 °C for 10 min. The beads were pelleted for 3 min at 300 ***g***, the supernatant was transferred to a new tube, and 100 mm DTT was added. The eluted samples were boiled for 5 min, and the content of the samples was analyzed by western blotting. Anti‐ATF4 mouse monoclonal and anti‐FAM175B mouse polyclonal antibodies were used to detect proteins immunoprecipitated with the anti‐ATF4 antibody. Anti‐FAM175B rabbit polyclonal and anti‐ATF4 rabbit monoclonal antibodies were used to detect proteins immunoprecipitated with the anti‐HAtag antibody.

Proteins for conventional western blotting were obtained by lysing cells in RIPA lysis buffer (50 mm Tris/HCl, 150 mm NaCl, 1% NP‐40, 0.5% sodium deoxycholate, and 0.1% SDS) and disruption with gentle sonication (on 3 s/off 3 s for 10 cycles). The protein concentration was confirmed by a bicinchoninic acid Protein Assay Kit (Thermo Fisher, Waltham, MA, USA) and was normalized with 5× loading buffer and ddH_2_O. Denatured total protein was separated by 10% SDS/PAGE and then transferred to nitrocellulose membranes. Nonspecific binding sites were blocked for 2 h at room temperature using 5% (w/v) milk (skim milk powder in TBST). The proteins were incubated overnight in 4 °C with the primary antibody. After washing six times with TBST, the blot was incubated with the HRP‐conjugated secondary antibody for 1 h at room temperature and visualized with an enhanced chemiluminescence system (Bio‐Rad, Hercules, CA, USA).

### 2.8 Immunofluorescence staining

Slides with cells were washed twice with PBS, fixed in 4% PFA for 15 min, and incubated in 0.25% Triton X‐100 for 10 min to rupture the cell membranes. The samples were blocked for 1 h using 1% BSA in PBST. The cells were incubated overnight at 4 °C in a mixture of 1 : 50 anti‐FAM175B (mouse polyclonal antibody; Abcam) and 1 : 50 anti‐ATF4 (rabbit monoclonal antibody; D4B8, CST). Next, the slides were washed three times with PBS for 5 min each and incubated for 1 h at room temperature in a mixture of fluorescent secondary antibodies (Alexa Fluor 488 goat anti‐mouse IgG, 1 : 100, Life Technologies (Waltham, MA USA); Alexa Fluor 568 goat anti‐rabbit IgG, 1 : 100; Life Technologies) in the dark. Then, the slides were washed three times with PBS, washed once with ddH_2_O, stained with DAPI (sc‐24941; Santa Cruz Biotechnology, Dallas, TX, USA), and imaged using a confocal microscope (IX83, FLUOVIEW FV1200; Olympus, Tokyo, Japan).

### 2.9 Cycloheximide test and ubiquitination assay

EC9706 and KYSE30 cells were transfected with pCMV‐HA or pCMV‐FAM175B. After 48 h of transfection, the cells were incubated with 0.1 mm cycloheximide (CHX) for 0, 0.5, 1, and 2 h. Then, the corresponding proteins were extracted for western blotting to assess ATF4 expression. In KYSE30 cells, ATF4 protein gray values were detected in 0, 0.5, 1, 1.5, 2, and 2.5 h after CHX treatment and divided by the corresponding GAPDH gray value. ATF4 protein expression level was shown as the percentage of 0 h and degradation curve was drawn as log 0 h%, and half‐life was confirmed by the corresponding time of log 50%. For the ubiquitination assay, EC9706 cells were seeded into a 100‐mm dish and were transfected with the pCMV‐FAM175B at 70% confluence. After 44 h, 20 μm MG132 (Cat No: S2619; Selleckchem, Houston, TX, USA) was added, and the cells were incubated at 37 °C with 5% CO_2_ for 4 h. The protein extraction process was the same as that used in the immunoprecipitation experiment, except for the inclusion of a mild ultrasonication step before centrifugation. The lysates were precleared, and the protein concentration in the lysates was quantified. Total protein (1.5 mg) was immunoprecipitated with anti‐ATF4 or anti‐IgG antibodies. The immunoprecipitated proteins were analyzed by conventional western blotting on 8% SDS/PAGE gels and incubated with an anti‐ubiquitin antibody to evaluate the ubiquitination level. ATF4 expression was assessed in the quantified input of each group; GAPDH expression was also assessed as the internal reference.

### 2.10 Luciferase gene reporter assay

The pGL3 plasmid carrying the CHOP promoter region [from −2000 base pairs to the transcription start site (TSS)] and the luciferase reporter gene were purchased from YouBio (Changsha, China). A total of 5 × 10^4^ EC9706 cells were seeded into 24‐well plates and cotransfected with 2 μg of pCMV‐FAM175B and/or 2.5 μL of si‐ATF4, 1 μg of the luciferase reporter plasmid, and 200 ng of the pRL‐TK plasmid using Lipofectamine 3000 according to the manufacturer's instructions. After the cells were cultured at 37 °C for 48 h, luciferase activity was assessed using a Dual Luciferase Assay Kit (Thermo Fisher); all procedures were performed according to the protocol provided by Thermo Fisher. Luciferase activity values were measured and normalized to the corresponding Renilla luciferase activity values.

### 2.11 RNA extraction and quantitative real‐time PCR (q‐PCR)

Total RNA was extracted using TRIzol (Invitrogen, Waltham, MA, USA) from two ESCC cell lines according to the manufacturer's protocol. Random primers were used for reverse transcription. Quantitative PCR was performed using SYBR‐Green mix (Invitrogen) and run in a 7500 Real‐Time PCR Systems (Applied Biosystems, Waltham, MA, USA). The amplification conditions were as follows: 95 °C for 2 min followed by 40 cycles of 95 °C for 15 s, 56 °C for 20 s, and 72 °C for 30 s and then followed by 72 °C for 2 min. GAPDH was used for internal reference. The RNA copy numbers were analyzed by the 2‐ΔΔCq method and compared it with housekeeping gene. Primer sequences used for amplification of the CHOP were 5′‐AGAACAGCCGTTACTTCAGGA (forward) and 5′‐CCGCTGGTAGGAGGTTTTAGAG (reverse); primer sequences for amplification of the GAPDH were 5′‐GGAGCGAGATCCCTCCAAAAT (forward) and 5′‐GGCTGTTGTCATACTTCTCATGG (reverse).

### 2.12 Statistical analysis

spss 19.0 (IBM, Armonk, NY, USA) for Windows was used for statistical analysis. Data are presented as the means ± standard deviations (SDs). Student's independent *t*‐test was used for statistical comparisons between the experimental and control groups. Student's paired *t*‐test was used for statistical comparisons between paired ESCC and noncancerous tissues. Log‐rank tests and Kaplan–Meier plots were applied to assess and show the difference in overall survival (OS) between subgroups. *P*‐values of < 0.05 were considered statistically significant.

## 3 Results

### 3.1 FAM175B expression is downregulated in ESCC tissues

**Figure 1:**
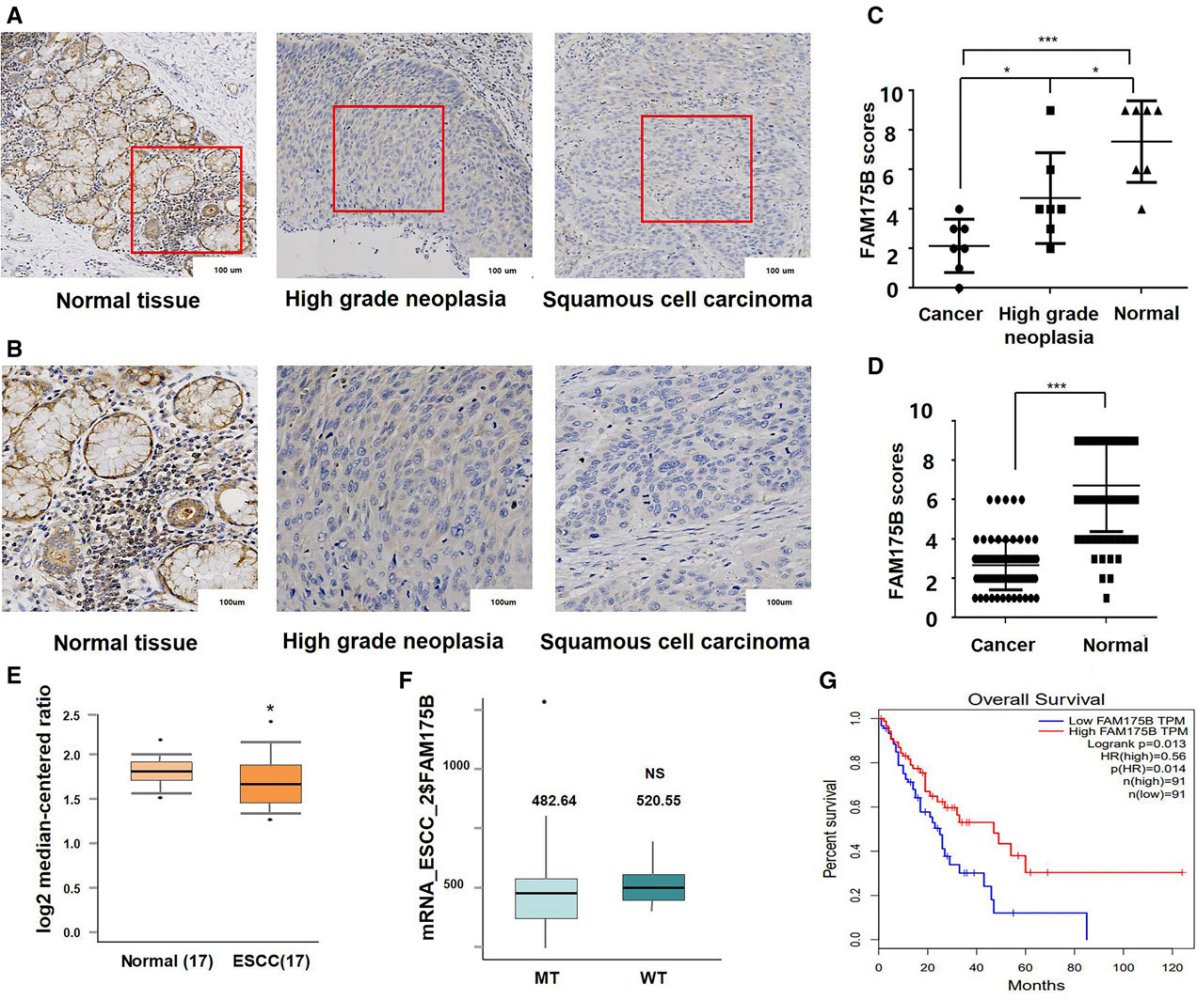
FAM175B expression is downregulated in ESCC and HGIEN tissues Representative IHC images are shown: strong staining of FAM175B in normal esophageal tissues, intermediate staining of FAM175B in esophageal HGIEN tissues, and weak staining of FAM175B in ESCC tissues (A). (B) Magnified images corresponding to those in (A). (C) Comparison of expression scores between 7 ESCC specimens and their paired adjacent HGIEN and noncancerous tissues. FAM175B expression was downregulated in HGIEN tissues (*t* = 2.44, *P* = 0.031) and ESCC tissues (*t* = 5.664, *P* < 0.001). (D) Analysis of expression scores for 75 pairs of ESCC and noncancerous tissues. FAM175B expression was also downregulated in the ESCC tissues (*t* = 13.27, *P* < 0.001). GEO database analysis (GSE20347) shows FAM175B mRNA was downregulated in ESCC compared with normal esophageal tissue (E). TCGA ESCC database analysis shows p53 mutation has no effect on FAM175B expression (F). Survival analysis in TCGA database shows higher FAM175B expression level had longer OS than others (G). The values are presented as the means ± SDs, with at least *n* = 3 per group. Statistical comparisons were analyzed by *t*‐test. *P* values < 0.05 were considered statistically significant. *P < 0.05; ***P < 0.001.

To evaluate FAM175B expression in ESCC, we performed immunohistochemical analysis on seven ESCC specimens and their paired adjacent HGIEN and noncancerous tissues (Fig. 1A,B). Compared with noncancerous tissues, both HGIEN tissues (*t* = 2.44, *P* = 0.031) and ESCC tissues (*t* = 5.664, *P* < 0.001) exhibited downregulated FAM175B expression. Moreover, compared with HGIEN tissues, ESCC tissues exhibited downregulated FAM175B expression (*t* = 2.41, *P* = 0.033, Fig. 1C). In the tissue array analysis of an independent and much larger cohort comprising 75 pairs of ESCC and noncancerous tissues, FAM175B expression was significantly downregulated in the ESCC group (*t* = 13.27, *P* < 0.001, Fig. 1D). In the correlation analysis between FAM175B expression and clinical variables in 75 ESCC patients, despite no significant difference, the patients with negative expression of FAM175B tend to have worse pathological grades and TNM stages (Table S1). Moreover, GEO (Gene Expression Omnibus) database analysis (GSE20347) also revealed the mRNA of FAM175B was downregulated in ESCC (*t* = −2.076, *P* = 0.025, Fig. 1E). In TCGA database which contains 96 cases of ESCC with p53 mutation information, we found the p53 mutation rate is as high as 90.6% (Table S10). Meanwhile, no influence of p53 mutation on FAM175B expression was observed (*t* = −0.998, *P* = 0.3377; Fig. 1F). In addition, survival analysis with 182 esophageal carcinoma patients in TCGA database indicated that higher FAM175B expression level had longer OS than others (Fig. 1G).

### 3.2 FAM175B suppresses cell growth in ESCC cell lines

**Figure 2:**
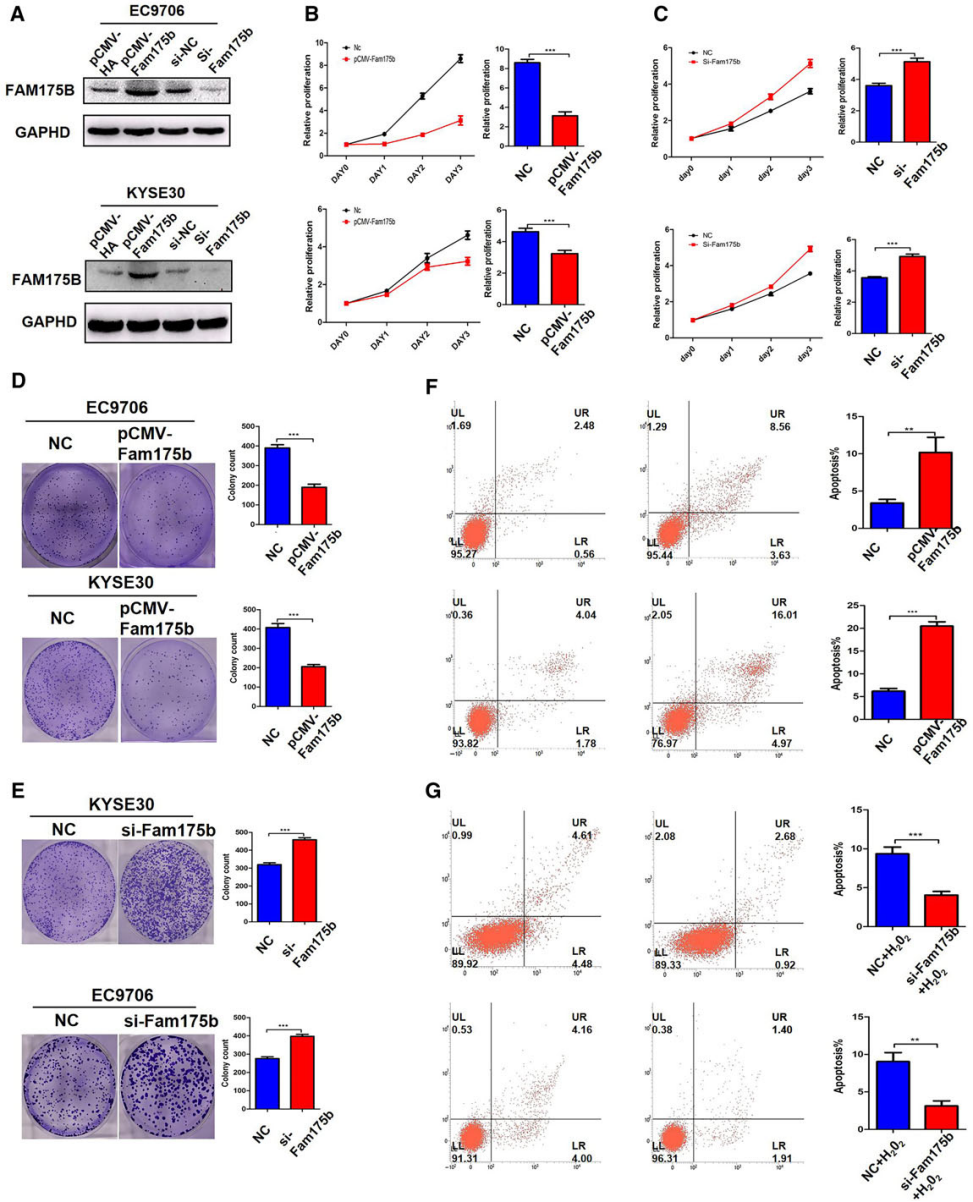
FAM175B suppresses proliferation and promotes apoptosis in ESCC cell lines. The western blotting results revealed that FAM175B was efficiently overexpressed and knocked down in KYSE30 and EC9706 cells (A). Relative rate of cell proliferation with FAM175B overexpression in KYSE30 (upper panel) and EC9706 (lower panel; B). Relative rate of cell proliferation with FAM175B knockdown in KYSE30 (upper panel) and EC9706 (lower panel; C). Colony formation assays of KYSE30 (upper panel) and EC9706 cells (lower panel) with FAM175B overexpression (D). Colony formation assays of KYSE30 (upper panel) and EC9706 cells (lower panel) with FAM175B knockdown (E). Flow cytometric apoptosis assays with FAM175B overexpression in EC9706 (upper panel) and KYSE30 cells (lower panel; F). Flow cytometric apoptosis assays with FAM175B knockdown in EC9706 (upper panel) and KYSE30 cells (lower panel) which treated with 500 uM H_2_O_2_ to induce apoptosis (G). All assays were performed in triplicate, and one representative result is displayed. The values are presented as the means ± SDs, with at least *n* = 3 per group. Statistical comparisons were analyzed by *t*‐test. *P* values < 0.05 were considered statistically significant. **P < 0.01; ***P < 0.001.

To determine the effects of FAM175B on cell proliferation, FAM175B was separately overexpressed and knocked down in both KYSE30 and EC9706 cells. The efficiency of overexpression and knockdown was confirmed via western blotting (Fig. 2A). With increased expression of FAM175B, the proliferation rate decreased and colony formation was inhibited in both cell lines. In the MTS assay, overexpression of FAM175B led to a 63.58% decrease in cell proliferation in KYSE30 cells (*t* = 20.60, *P* < 0.001, Fig. 2B upper panel, Table S6) and a 29.96% decrease in EC9706 cells (*t* = 9.162, *P* < 0.001, Fig. 2B lower panel, Table S7). Knockdown of FAM175B showed the opposite results in KYSE30 cells (*t* = 11.62, *P* < 0.001, Fig. 2C upper panel, Table S8) and EC9706 cells (*t* = 16.32, *P* < 0.001, Fig. 2C lower panel, Table S9). In the colony formation assay, overexpression of FAM175B led to a 47.92% decrease in the number of foci in KYSE30 cells (*t* = 18.92, *P* < 0.001, Fig. 2D lower panel) and a 49.59% decrease in EC9706 cells (*t* = 14.75, *P* < 0.001, Fig. 2D upper panel). Knockdown of FAM175B showed the opposite results in KYSE30 cells (*t* = 15.72, *P* < 0.001, Fig. 2E upper panel) and EC9706 cells (*t* = 14.42, *P* < 0.001, Fig. 2E lower panel).

### 3.3 FAM175B promotes cell apoptosis

**Figure 3:**
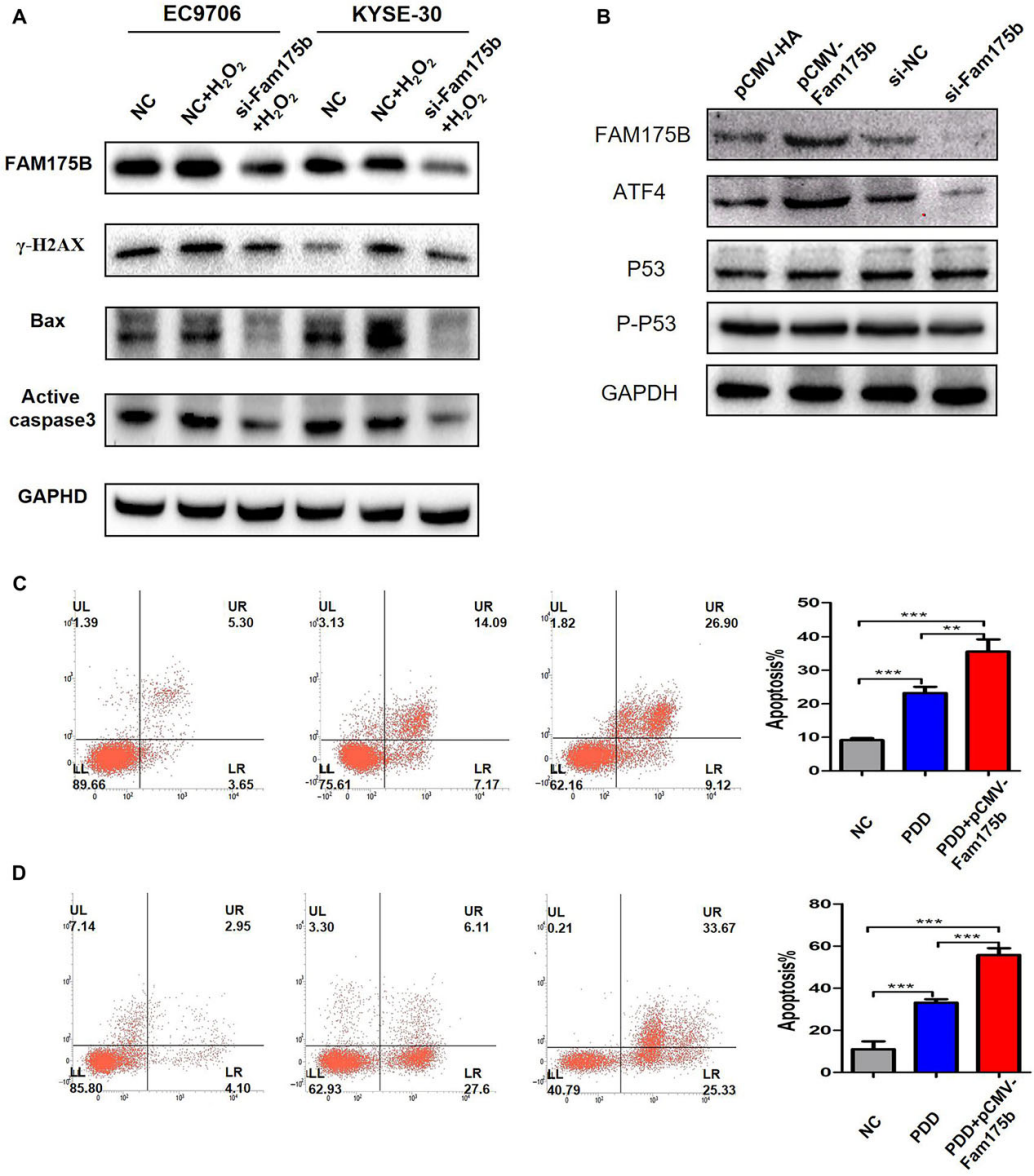
FAM175B induces cell apoptosis in a p53‐independent manner and the synthetic proapoptotic effect with cisplatin. H_2_O_2_ treatment significantly upregulated apoptosis‐related proteins such as Bax, active caspase‐3, and γ‐H2AX. However, the combined treatment of si‐FAM175B attenuated the activation of these proapoptotic proteins (A). When FAM175B was overexpressed or knocked down in KYSE30 cell, the expression of ATF4 was upregulated or decreased accordingly without change of p53 expression or p53 phosphorylation level (B). Overexpression of FAM175B in EC9706 (C) and KYSE30 (D) synergistically enhanced the pro‐apoptotic effect of cisplatin. The values are presented as the means ± SDs, with at least *n* = 3 per group. Statistical comparisons were analyzed by *t*‐test. *P* values < 0.05 were considered statistically significant. **P < 0.01; ***P < 0.001.

To investigate the effects of FAM175B on tumor apoptosis, si‐FAM175B and pCMV‐FAM175B were used to alter FAM175B expression in two ESCC cell lines. Overexpression of FAM175B induced apoptosis rate significantly increased in both EC9706 cells (*t* = 5.647, *P* = 0.0048, Fig. 2F upper panel) and KYSE30 cells (*t* = 21.97, *P* < 0.0001, Fig. 2F lower panel) compared with that in the control group. In the other hand, knockdown of FAM175B attenuated H_2_O_2_‐induced cell apoptosis in both EC9706 cells (*t* = 9.096, *P* = 0.0008, Fig. 2G upper panel) and KYSE30 cells (*t* = 7.435, *P* = 0.0017, Fig. 2G lower panel) compared with that in the control group. Generally, FAM175B had an important proapoptotic role on ESCC cells. Moreover, FAM175B knockdown attenuated H_2_O_2_‐induced upregulation of proapoptotic proteins (such as Bax, active caspase‐3, and γ‐H2AX, Fig. 3A), but cell cycle‐related proteins (p21, p27, cyclin d1, and CDK4) were not found to have significant changes (data not shown). In addition, the combination of cisplatin treatment and FAM175B overexpression in two ESCC cell lines showed a synergistic proapoptotic effect (Fig. 3C,D).

### 3.4 FAM175B colocalizes and interacts with ATF4 in ESCC cells

**Figure 4:**
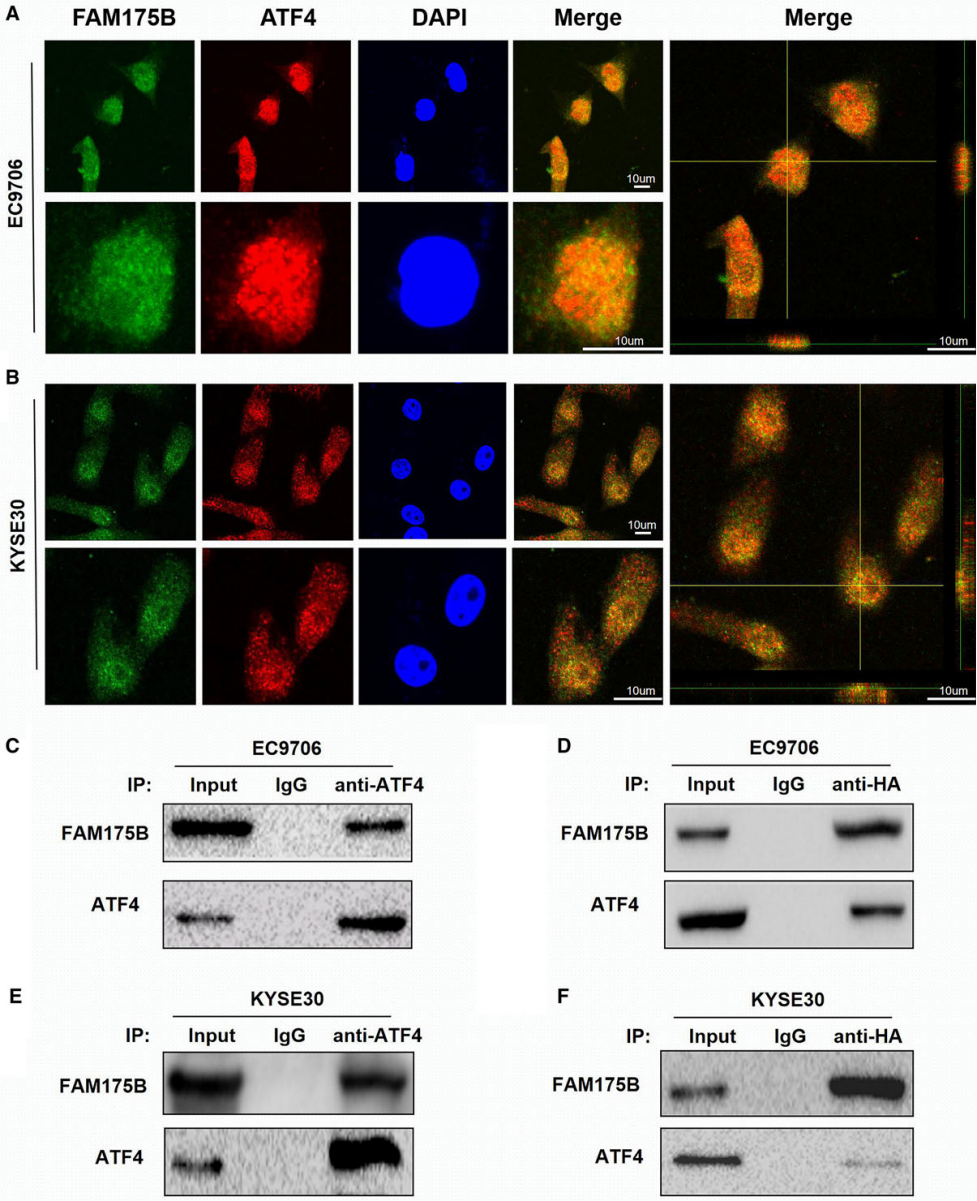
FAM175B colocalizes and interacts with ATF4 in ESCC cells. FAM175B and ATF4 were labeled with the corresponding primary antibodies and incubated with fluorescent secondary antibodies. Confocal microscopy was used to detect colocalization signals. The appearance of obvious merged puncta validated the colocalization of FAM175B and ATF4 in the cytoplasm and nucleus of EC9706 (A) and KYSE30 (B) cells (blue: DAPI; green: FAM175B; red: ATF4). pCMV‐FAM175B was transfected, and reciprocal co‐IP assays were performed in EC9706 (C,D) and KYSE30 (E,F) cells. Three samples were pipetted into three different lanes according to the antibodies added to or absent from the samples (input group: no antibody; IgG group: IgG; ATF4 group: anti‐ATF4 antibody; FAM175B group: anti‐HAtag antibody). The length of the scale bars is 4 mm in upper panel of figure A and 20 mm in lower panel and 12 mm in 3D merged image; length of the scale bars in the upper panel of figure B is 4 mm, and the length of the lower panel and 3D merged image is 12 mm.

A previous study reported that FAM175B interacts with transcription factors of the AP‐1 family, suggesting that FAM175B can participate in tumorigenesis in a p53‐independent manner. We first performed western blotting to investigate the regulatory association between FAM175B and ATF4, an AP‐1 family member. We found that ATF4 expression was upregulated in the FAM175B overexpression group and downregulated in the knockdown group. The expression and phosphorylation levels of p53 (Ser15) were also assessed in these groups, but no difference of these forms of p53 in either group was observed (Fig. 3B). Then, we performed immunofluorescence assays and revealed that FAM175B colocalized with ATF4 in both the cytoplasm and nucleus in EC9706 and KYSE30 cells (Fig. 4A,B). To further prove this interaction, we performed reciprocal coimmunoprecipitation (co‐IP) assays in these two ESCC cell lines; these results also indicated that both exogenous and endogenous FAM175B could interact with ATF4 (Fig. 4C–F).

### 3.5 FAM175B can inhibit ubiquitination‐dependent ATF4 degradation

**Figure 5:**
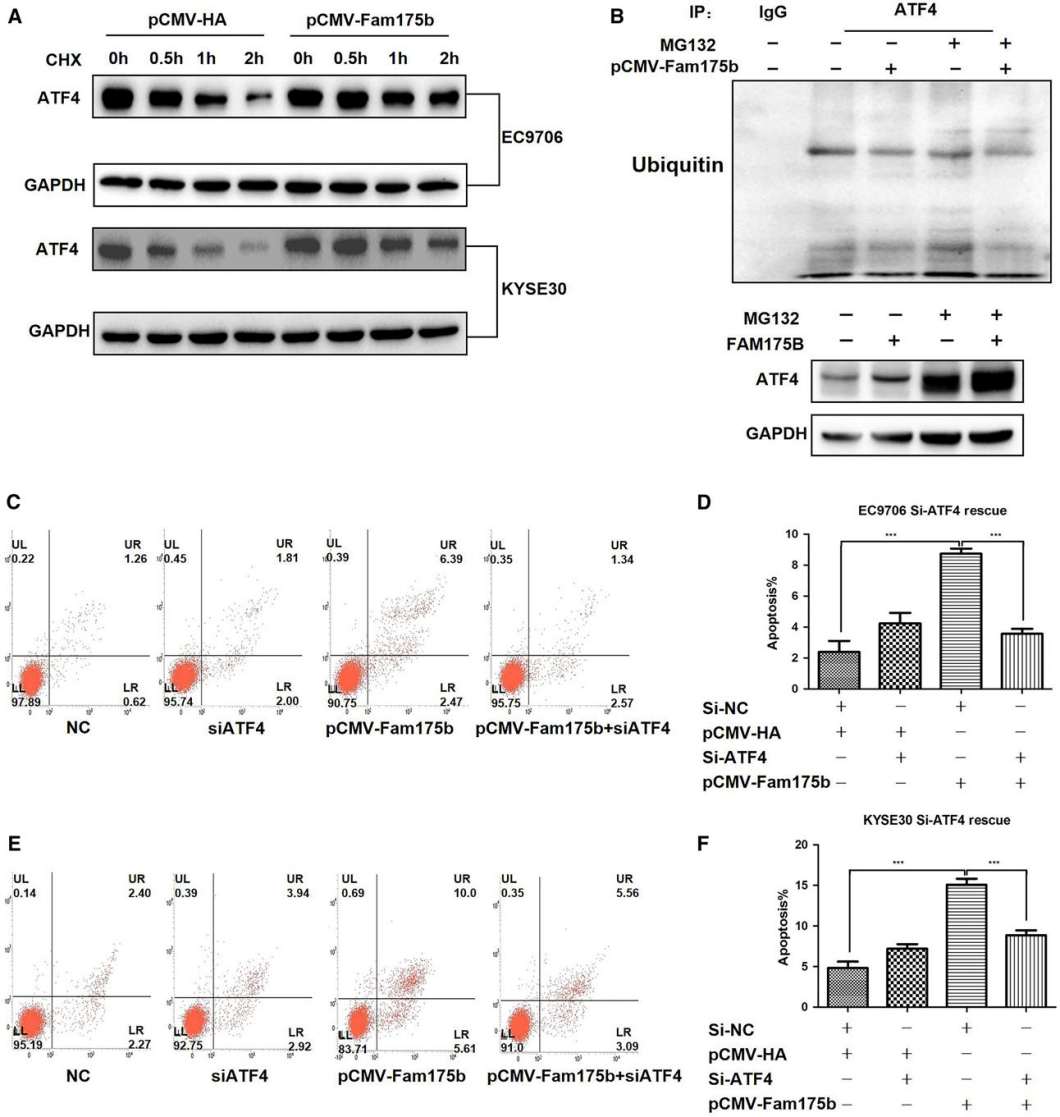
FAM175B promotes cell apoptosis by inhibiting ubiquitination‐dependent ATF4 degradation. pCMV‐HA‐ or pCMV‐FAM175B‐transfected EC9706 and KYSE30 cells were incubated with 0.1 mm CHX for 0, 0.5, 1, and 2 h, and the ATF4 expression level was assessed via western blotting. FAM175B overexpression reduced the rate of ATF4 degradation (A). After transfection with pCMV‐HA or pCMV‐FAM175B, cells were incubated with 20 μm MG132 for 4 h, and 1.5 mg of the lysate was then incubated overnight with rabbit IgG or anti‐ATF4 and protein A/G agarose beads. The ATF4 ubiquitination level was assessed via conventional western blotting on 8% SDS/PAGE gels. FAM175B overexpression significantly reduced the ATF4 ubiquitination level and increased the ATF4 protein level (B). pCMV‐FAM175B and/or si‐ATF4 was transfected into two ESCC cell lines, and the apoptosis rate was assessed. ATF4 knockdown rescued the increased apoptosis rate induced by FAM175B overexpression in EC9706 (C,D) and KYSE30 cells (E,F). All assays were performed in triplicate, and one representative result is displayed. The values are presented as the means ± SDs, with at least *n* = 3 per group. Statistical comparisons were analyzed by *t*‐test. *P* values < 0.05 were considered statistically significant. ***P < 0.001.

According to the ubiquitin hydrolase role of the BRISC enzyme complex, we speculated that FAM175B can inhibit ATF4 ubiquitin labeling and subsequent proteasomal degradation through interacting with ATF4. To confirm this hypothesis and provide more evidence to reveal the related mechanism, we first performed CHX‐treated ATF4 degradation test and confirmed the half‐time of ATF4 protein (Table S2), which showed that when FAM175B was overexpressed in two ESCC cell lines, the degradation rate of ATF4 decreased compared to that in the control group (Fig. 5A). This result suggested that FAM175B can inhibit ATF4 protein degradation. Next, we treated EC9706 cells with MG132 to detect the level of ATF4 ubiquitination. Protein in the cell lysates was quantified, and immunoprecipitation with the anti‐ATF4 antibody was performed on 1.5 mg of protein from each group. The western blotting results showed that FAM175B can reduce the level of ATF4 ubiquitin labeling and increase the protein level of ATF4 (Fig. 5B).

### 3.6 FAM175B promotes cell apoptosis via the ATF4‐CHOP pathway

**Figure 6:**
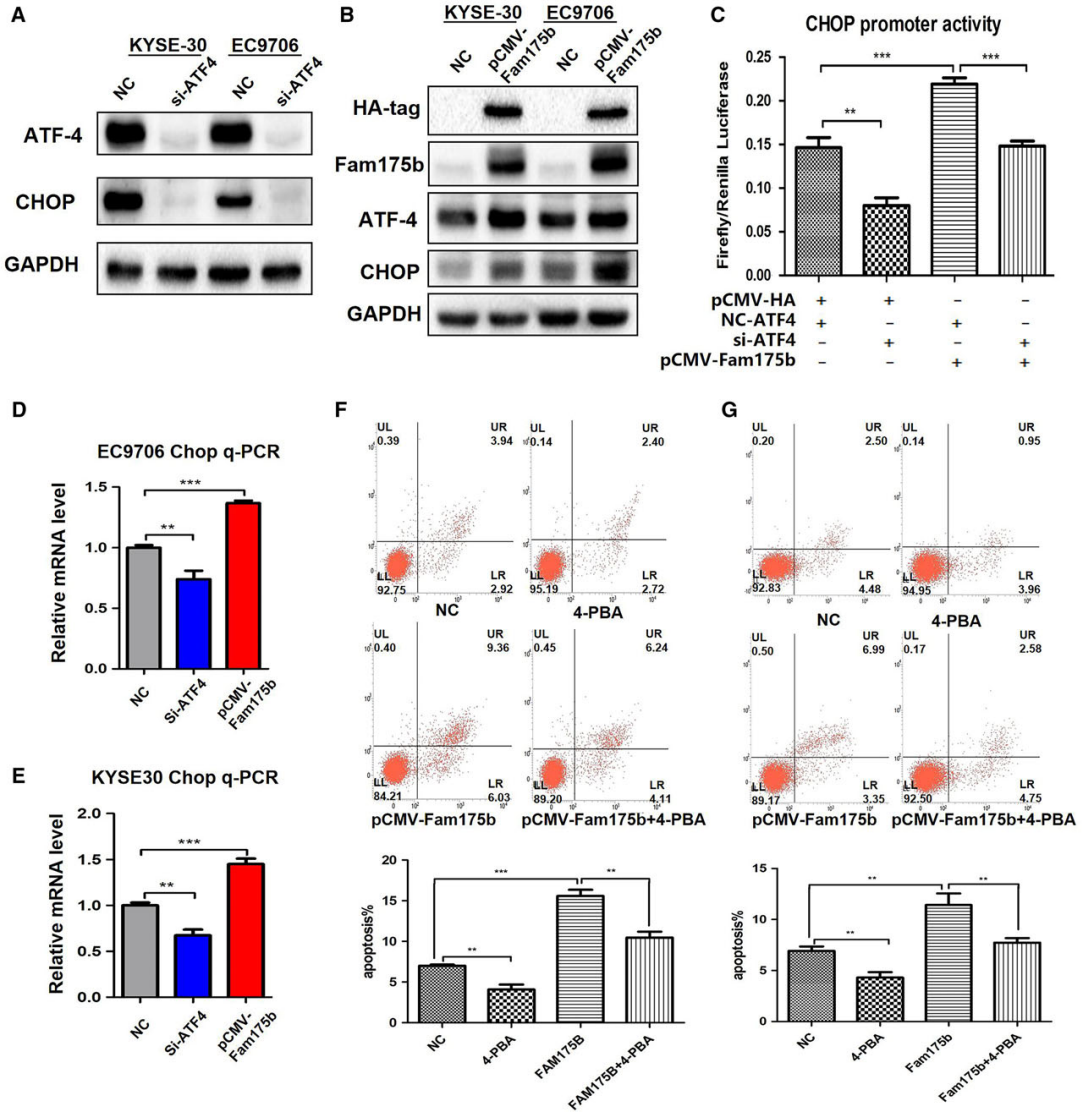
FAM175B promotes cell apoptosis via the ATF4‐CHOP pathway. The expression of the ATF4 downstream gene CHOP was assessed by western blotting; ATF4 knockdown significantly reduced CHOP expression levels(A), while FAM175B overexpression promoted CHOP expression by increasing ATF4 levels (B). In luciferase reporter gene assay, EC9706 cells were cotransfected with pCMV‐FAM175B, si‐ATF4, and the pGL3 vector carrying the CHOP promoter region. A Renilla luciferase plasmid was simultaneously transfected as an internal reference. The results show that FAM175B can induce CHOP promoter activity in an ATF4‐dependent manner (C). q‐PCR test shows si‐ATF4 treatment reduced CHOP mRNA levels, while overexpression of FAM175B upregulated CHOP mRNA levels in EC9706 (D) and KYSE30 (E) cell lines. Twenty‐four hours after cell transfection, 5 mm 4‐PBA was added to the medium for another 24 h. The results show that the apoptosis rate induced by FAM175B overexpression was rescued by 4‐PBA treatment in KYSE30 (F) and EC9706 (G) cells. All assays were performed in triplicate, and one representative result is displayed. The values are presented as the means ± SDs, with at least *n* = 3 per group. Statistical comparisons were analyzed by *t*‐test. *P* values < 0.05 were considered statistically significant. **P < 0.01; ***P < 0.001.

As ATF4 plays a key role in one of the pathways of ERS‐related apoptosis, to further explore the mechanism of apoptosis induced by FAM175B, we overexpressed FAM175B and/or transfected si‐ATF4 in KYSE30 and EC9706 cells and then assessed the cell apoptosis rate. Knockdown of ATF4 expression with si‐ATF4 rescued the increase in the apoptosis rate induced by FAM175B overexpression from 8.38% to 3.57% (*t* = 15.630, *P* < 0.0001) in EC9706 cells and from 15.08% to 8.85% (*t* = 11.24, *P* = 0.0004) in KYSE30 cells (Fig. 5C–F). Then, the expression of the ATF4 downstream protein CHOP was assessed through western blotting. We found that si‐ATF4 reduced the CHOP expression level and that FAM175B overexpression resulted in an increased level of CHOP (Fig. 6A,B). Moreover, consistent with the results of the western blot analysis, the q‐PCR analysis results showed that ATF4 knockdown downregulated CHOP mRNA expression and FAM175B overexpression upregulated CHOP mRNA expression (Fig. 6D,E, Table S4 and S5).

Luciferase gene reporter experiments were also performed. FAM175B overexpression in EC9706 cells induced significant transcriptional activation of CHOP, and these effects were rescued when si‐ATF4 was transfected into EC9706 cells (Fig. 6C, Table S3). To further demonstrate that the proapoptotic effect of FAM175B is mediated through the ATF4‐CHOP pathway, we treated cells with the CHOP inhibitor 4‐PBA (5 mm) and found that 4‐PBA can rescue the elevated apoptosis rate induced by FAM175B overexpression in KYSE30 (Fig. 6F) and EC9706 (Fig. 6G) cells.

## 4 Discussion

FAM175B expression has been reported to be downregulated in several cancers such as liver cancer, breast cancer, and renal cancer (Zhang *et al*., 2014). FAM175B expression can be induced by DNA damage and can antagonize the ubiquitination of p53 to perform its tumor‐suppressive function (Zhang *et al*., 2014). Available evidence suggests that FAM175B can suppress tumorigenesis in a p53‐dependent manner, but the role of FAM175B in ESCCs, almost 70% of which are p53‐mutated, remains unexplored.

Our study showed significant downregulation of FAM175B expression in ESCC tissues and esophageal HGIEN tissues; meanwhile, worse pathological grades and TNM stages were observed in ESCC patients with negative expression of FAM175B. Moreover, GEO database analysis (GSE20347) also showed the mRNA of FAM175B was downregulated in ESCC. By analyzing the TCGA database which contains 182 samples, we found esophageal carcinoma patients with higher FAM175B expression level had longer OS. These evidences suggest that FAM175B may have a function in suppressing ESCC carcinogenesis. Due to the high mutation rate of p53 in ESCC, this tumor suppressor effect may be p53‐independent. The ESCC cell lines EC9706 and KYSE30, both of which carry p53 mutations, were selected for further assays. Then, MTS, colony formation and flow cytometric apoptosis assays were conducted to clarify the tumor‐suppressive role of FAM175B, and these results showed that FAM175B can inhibit cell proliferation and colony formation and promoted apoptosis in a p53‐independent manner. Nearly half of all cancers are p53‐mutated (Liu *et al*., 2013; Stokłosa and Gołąb, 2005; Szymañska and Hainaut, 2003), suggesting that the p53‐independent tumor‐suppressive effect of FAM175B in ESCC also exists in other cancer types with p53 mutations. According to the significant downregulation of FAM175B expression in ESCC and HGIEN tissues, we suggested that FAM175B has great potential as a biomarker for early diagnosis and prognosis in ESCC. Moreover, the western blot results showed that FAM175B knockdown attenuated H_2_O_2_‐induced activation of proapoptotic proteins and FAM175B overexpression enhanced cisplatin‐induced cell apoptosis in EC9706 and KYSE30 cells, these findings revealed that FAM175B downregulation and the absence of activity in relevant pathways may play a role in chemotherapy drug resistance; thus, molecular drugs targeting FAM175B‐related pathways may have important clinical value in combating the antiapoptotic property of tumor cells.

**Figure 7:**
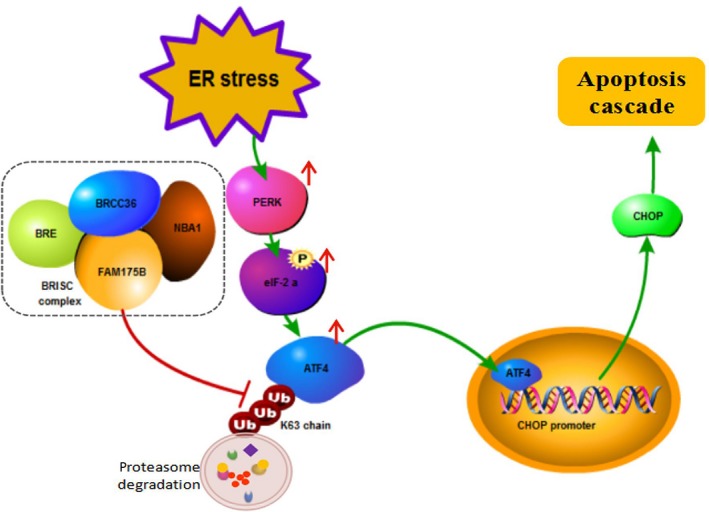
FAM175B inhibits ubiquitination‐dependent ATF4 degradation and promotes cell apoptosis. Schematic diagram of the mechanism by which FAM175B promotes apoptosis: Stimulating factors such as hypoxia can induce cellular ERS, and persistent ERS activates the PERK‐eIF‐2α‐ATF4 pathway. As a transcriptional activator, the upregulated ATF4 protein enters the nucleus and promotes the transcription and expression of CHOP, which in turn induces an apoptotic cascade. As a member of the BRISC ubiquitin hydrolase complex, FAM175B mediates ERS‐related apoptosis by directly interacting with ATF4 and inhibiting its ubiquitin‐dependent proteasomal degradation.

Hypoxia is a salient feature of the tumor microenvironment; it can inhibit the correct folding of endoplasmic reticulum proteins to induce ERS and the UPR (Koumenis *et al*., 2002). Persistent ERS and activation of the UPR disturbs endoplasmic reticulum homeostasis and cause the transition to cell apoptosis for cytoprotection; however, cancer cells can overcome the extreme hypoxia‐induced proapoptotic effect and continuously proliferate and metastasize (Bobrovnikova‐Marjon *et al*., 2010; Ma and Hendershot, 2004; Wang *et al*., 2014). Previous studies have shown that FAM175B interacts with three members of the AP‐1 family: the ATF4, ATF5, and JunD proteins. These findings suggest that FAM175B not only recruits members of the BRISC enzyme complex but also can interact with other transcription factors to influence downstream gene expression and cancer development. According to the deubiquitination effect of FAM175B and the key role of ATF4 in the protein kinase RNA‐like ER kinase (PERK)‐ and phosphorylated eukaryotic initiation factor 2 alpha‐related ERS pathways, we hypothesize that FAM175B can inhibit the ubiquitination level of its interacting protein ATF4 and increase the ATF4 protein level to promote ERS‐induced apoptosis and that the downregulation of FAM175B expression facilitates apoptosis resistance and tumorigenesis (Fig. 7).

To verify this hypothesis, we first confirmed the colocalization and interaction of the FAM175B and ATF4 proteins by confocal microscopy and protein co‐IP assay. Furthermore, by CHX and ubiquitination assays, we proved that FAM175B can inhibit ATF4 degradation by reducing the level of ATF4 ubiquitin labeling. In addition, we found that si‐ATF4 transfection rescued the increased apoptosis rate induced by FAM175B overexpression, indicating that FAM175B can promote cell apoptosis, at least in part, in an ATF4‐dependent manner. To further explore the possible underlying mechanism, we subcloned the CHOP promoter region (from −2000 base pairs to the TSS) into the pGL3 plasmid and performed a CHOP promoter activity assay. Meanwhile, related western blot analysis and q‐PCR test were performed. We found that the overexpression of FAM175B can elevate CHOP expression at both the transcriptional and translational levels; in contrast, ATF4 knockdown reduced CHOP expression. Moreover, as a chemical molecular chaperone that can stabilize protein conformations and improve the folding capacity of the endoplasmic reticulum, 4‐PBA was used in an apoptosis rescue assay to antagonize the response of the CHOP pathway. The results showed that 4‐PBA treatment significantly reduced cell apoptosis induced by FAM175B overexpression in EC9706 and KYSE30 cells, indicating that FAM175B can affect apoptosis by regulating the ATF4‐CHOP pathway. Hypoxic conditions can increase the therapeutic resistance of tumor cells due to the limitations of chemotherapy drug diffusion and reduced production of oxide free radicals by radiotherapy and chemotherapy (Graeber *et al*., 1996; Majsterek *et al*., 2004; Teicher, 1995). The results of our study suggest that the lack of FAM175B‐ATF‐4‐CHOP pathway activity may facilitate the development of apoptosis resistance in cancer cells in a hypoxic environment. Further study of the means by which tumor cells adapt to persistent hypoxia‐induced ERS and antiapoptotic mechanisms will help to overcome the limitations of current antitumor therapies. In summary, our study verified that FAM175B can participate in ERS‐related apoptosis by regulating the ATF‐4‐CHOP pathway and that FAM175B can suppress tumorigenesis in p53‐mutated ESCC. These findings provide new insight into the biological role of FAM175B in tumorigenesis and its value as biomarker for the early diagnosis of ESCC.

## 5 Conclusions

FAM175B expression is significantly downregulated in HGIEN and ESCC tissues. Exogenous expression of FAM175B can suppress the proliferation of ESCC cells mainly by increasing apoptosis rate. Through immunofluorescence confocal and co‐IP assay, we confirmed that ATF4, one transcriptional activator, colocalized and interacted with FAM175B. FAM175B can inhibit the ubiquitination labeling of ATF4 and its proteasome degradation. Then, ATF4 can induce the transcription activation of CHOP to promote apoptosis. In this study, we revealed that FAM175B participates in the regulation of ERS‐related apoptosis and suppresses ESCC tumorigenesis in a p53‐independent manner.

## Conflict of interest

The authors declare no conflict of interest.

## Author contribution

SZ and LM conceived and designed the study. ZZ collected clinical samples. HCL and GQD completed bioinformatics analysis. SLG designed pCMV‐HA vector. YZ and YY performed all other experiments, statistical analysis, and wrote the paper. PL and S Zhu reviewed and edited the manuscript.

## Supporting information


**Fig. S1.** Positive control of p53 activation and ATF4 protein degradation curve.

**Table S1.** Associations between FAM175B expression and clinicopathological factors in 75 ESCC patients.
**Table S2.** ATF4 protein half‐time detection in KYSE30.
**Table S3.** CHOP promoter Luciferase report test raw results.
**Table S4.** EC9706 q‐PCR raw results.
**Table S5.** KYSE30 q‐PCR raw results.
**Table S6.** KYSE30 MTS raw results (overexpression).
**Table S7.** EC9706 MTS raw results (overexpression).
**Table S8.** KYSE30 MTS raw results (knockdown).
**Table S9.** EC9706 MTS raw results (knockdown).
**Table S10.** P53 mutation in TCGA database.
