# Supplementary material for: FAM175B promotes apoptosis by inhibiting ATF4 ubiquitination in esophageal squamous cell carcinoma
Source: Mol Oncol. 2019 Mar 23;13(5):1150–65. doi: 10.1002/1878-0261.12474 (PMC6487841; doi:10.1002/1878-0261.12474)
Supplement: Supplementary file 1 — Fig. S1. Positive control of p53 activation and ATF4 protein degradation curve. [file MOL2-13-1150-s001.pdf]

A

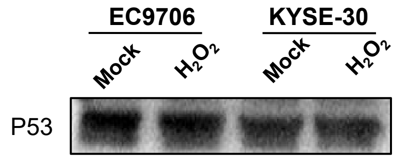

B

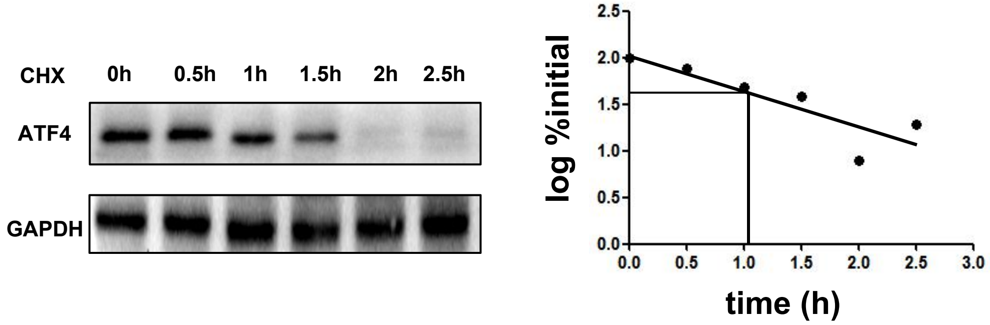

Supplementary Figure 1. Positive control of p53 activation and ATF4 protein degradation curve

EC9706 and KYSE30 cells were incubated with 500 uM H2O2 for 24h and p53 protein level was detected with western blot (A). ATF4 and GAPDH gray values were detected in 0h, 0.5 h, 1 h, 1.5, 2h, 2.5h after CHX treatment, then log 0h% was calculated and ATF4 protein degradation curve was drawn. ATF4 half-life was confirmed by the corresponding time of log 50% (B).
