# Supplementary material for: FAM175B promotes apoptosis by inhibiting ATF4 ubiquitination in esophageal squamous cell carcinoma
Source: Mol Oncol. 2019 Mar 23;13(5):1150–65. doi: 10.1002/1878-0261.12474 (PMC6487841; doi:10.1002/1878-0261.12474)
Supplement: Supplementary file 2 — Table S1. Associations between FAM175B expression and clinicopathological factors in 75 ESCC patients. Table S2. ATF4 protein half‐time detection in KYSE30. Table S3. CHOP promoter Luciferase report test raw results. Table S4. EC9706 q‐PCR raw results. Table S5. KYSE30 q‐PCR raw results. Table S6. KYSE30 MTS raw results (overexpression). Table S7. EC9706 MTS raw results (overexpression). Table S8. KYSE30 MTS raw results (knockdown). Table S9. EC9706 MTS raw results (knockdown). Table S10. P53 mutation in TCGA database. [file MOL2-13-1150-s002.pdf]

Supplementary Table 1. Associations between FAM175B expression and clinicopathological factors in 75 ESCC patients

Variables	FAM175B		χ2	*P-value*
	Positive	Negative		
**Gender**				
Male	27	26	0.861	0.3535
Female	13	8		
**Age（years）**				
≥65	13	13	0.4557	0.4996
<65	27	21		
**Tumor size（cm）**				
≥5	15	16	0.893	0.3447
<5	25	17		
**Pathological Stage**			0.1777	0.6734
I、II	27	22		
III、IV	13	13		
**Clinical Stage**				
1、2	22	12	3.232	0.0722
3、4	18	23		
**Lymph Node Metastasis**			1.5038	0.2201
Negtive	16	9		
positive	24	25		
**T stage**				
T1、T2	18	12	0.4803	0.4883
T3、T4	18	17		

Supplementary Table 2. ATF4 protein half-time detection in KYSE30

time		0h	0.5h	1.0h	1.5h	2.0h	2.5h
gray value	ATF4	22774.731	19067.418	14535.004	12087.004	2187.74	4377.004
	GAPDH	17775.974	19268.054	23509.296	24694.225	21504.447	21374.024
ATF4/GAPDH		1.281208613	0.989587117	0.618266238	0.489466829	0.101734306	0.204781467
0h%		100%	77.24%	48.26%	38.20%	7.94%	15.98%
log0h%		2	1.887842265	1.683587318	1.582063363	0.899820502	1.203576775

Supplementary Table 3. CHOP promoter Luciferase report test raw results

	NC		
Firefly	59451.2	62673.9	76274.1
Renilla	376832	446379	552483
Firefly/Renilla	0.157765795	0.140405127	0.138056918
	si-ATF4		
Firefly	18578.3	16642.6	24832.5
Renilla	213681	236273	298582
Firefly/Renilla	0.08694409	0.07043801	0.083168108
	pCMV-fam175b		
Firefly	42583.6	36734.3	41682.4
Renilla	188429	167513	196526
Firefly/Renilla	0.225992814	0.219292234	0.212096109
	si-ATF4+pCMV-fam175b		
Firefly	51738.1	91168.2	71692.8
Renilla	365831	598372	476838
Firefly/Renilla	0.141426232	0.152360405	0.150350433

Supplementary Table 4. EC9706 q-PCR raw results

Sample Name	Target Name	CT	CT Mean	ΔCT	ΔΔCT	2^（-ΔΔCT）
NC	GAPDH	20.92854309	20.93606377			
NC	GAPDH	20.95384407	20.93606377			
NC	GAPDH	20.92580605	20.93606377			
NC	CHOP-1	28.64650536	28.6588974	7.722833633	-0.012392044	1.008626506
NC	CHOP-1	28.69326782	28.6588974	7.722833633	0.034370422	0.976457784
NC	CHOP-1	28.63691521	28.6588974	7.722833633	-0.021982193	1.015353568
SI-ATF4	GAPDH	20.52105141	20.51085091			
SI-ATF4	GAPDH	20.5304718	20.51085091			
SI-ATF4	GAPDH	20.48103333	20.51085091			
SI-ATF4	CHOP-1	28.52304459	28.6716671	8.01219368	0.289360046	0.818264946
SI-ATF4	CHOP-1	28.71526146	28.6716671	8.204410553	0.48157692	0.716194369
SI-ATF4	CHOP-1	28.77668762	28.6716671	8.265836716	0.543003082	0.686340749
FAM175B	GAPDH	19.92880058	19.81596565			
FAM175B	GAPDH	19.8272171	19.81596565			
FAM175B	GAPDH	19.69188309	19.81596565			
FAM175B	CHOP-1	27.28720284	27.16263771	7.471237183	-0.251596451	1.190523791
FAM175B	CHOP-1	27.09392166	27.16263771	7.277956009	-0.444877625	1.361198649
FAM175B	CHOP-1	27.10679245	27.16263771	7.290826797	-0.432006836	1.349108927

Supplementary Table 5. KYSE30 q-PCR raw results

Sample Name	Target Name	CT	CT Mean	ΔCT	ΔΔCT	2^（-ΔΔCT）
NC	GAPDH	20.73852158	20.86878777			
NC	GAPDH	20.94121933	20.86878777			
NC	GAPDH	20.92662621	20.86878777			
NC	CHOP-1	28.88231659	28.88710785	8.018320084	-0.00479126	1.003326569
NC	CHOP-1	28.93295097	28.88710785	8.018320084	0.045843124	0.96872352
NC	CHOP-1	28.84605408	28.88710785	8.018320084	-0.041053772	1.028865055
SI-ATF4	GAPDH	20.03677177	20.08063698			
SI-ATF4	GAPDH	20.09820557	20.08063698			
SI-ATF4	GAPDH	20.10693169	20.08063698			
SI-ATF4	CHOP-1	28.52304459	28.6716671	8.442407608	0.424087524	0.745309976
SI-ATF4	CHOP-1	28.71526146	28.6716671	8.634624481	0.616304398	0.652339821
SI-ATF4	CHOP-1	28.77668762	28.6716671	8.696050644	0.67773056	0.625147894
FAM175B	GAPDH	19.63554573	19.70085716			
FAM175B	GAPDH	19.69820976	19.70085716			
FAM175B	GAPDH	19.76881218	19.70085716			
FAM175B	CHOP-1	27.24182854	27.48361206	7.540971375	-0.477348709	1.392182851
FAM175B	CHOP-1	27.1852047	27.48361206	7.484347534	-0.533972549	1.447910622
FAM175B	CHOP-1	27.12380486	27.48361206	7.422947693	-0.595372391	1.510862521

Supplementary Table 6. KYSE30 MTS raw results（overexpression）

raw			normalized		
day0	NC	pCMV-fam175b	day0	NC	pCMV-fam175b
	0.15405	0.14895		1.005056271	1.016723549
	0.15385	0.14525		1.003751427	0.991467577
	0.15275	0.14615		0.996574784	0.997610922
	0.15245	0.14565		0.994617518	0.994197952
AVERAGE	0.153275	0.1465			
day1	NC	pCMV-fam175b	day1	NC	pCMV-fam175b
	0.291	0.1669		1.898548361	1.139249147
	0.2967	0.1519		1.935736421	1.036860068
	0.289	0.1468		1.885499918	1.002047782
	0.2996	0.1528		1.954656663	1.043003413
day2	NC	pCMV-fam175b	day2	NC	pCMV-fam175b
	0.71085	0.25005		4.637742619	1.706825939
	0.79635	0.26595		5.19556353	1.815358362
	0.83825	0.29235		5.468928397	1.99556314
	0.94425	0.28845		6.160495841	1.96894198
day3	NC	pCMV-fam175b	day3	NC	pCMV-fam175b
	1.2922	0.3817		8.430598597	2.605460751
	1.2766	0.4707		8.328820747	3.212969283
	1.3933	0.4552		9.090197358	3.107167235
	1.3071	0.5264		8.527809493	3.593174061

Supplementary Table 7. EC9706 MTS raw results（overexpression）

	raw			normalized	
day0	NC	pCMV-fam175b	day0	NC	pCMV-fam175b
	0.35085	0.32275		0.988240265	0.998839458
	0.35105	0.32295		0.988803605	0.999458414
	0.35105	0.32265		0.988803605	0.998529981
	0.36715	0.32415		1.034152524	1.003172147
AVERAGE	0.355025	0.323125			
day1	NC	pCMV-fam175b	day1	NC	pCMV-fam175b
	0.5828	0.4496		1.641574537	1.391411992
	0.6066	0.4851		1.70861207	1.501276596
	0.6005	0.4945		1.691430181	1.530367505
	0.5781	0.482		1.628336033	1.491682785
day2	NC	pCMV-fam175b	day2	NC	pCMV-fam175b
	1.12395	0.89605		3.165833392	2.773075435
	1.31845	0.92265		3.713682135	2.855396518
	1.23415	0.98655		3.476234068	3.053152805
	1.14985	1.00075		3.238786001	3.097098646
day3	NC	pCMV-fam175b	day3	NC	pCMV-fam175b
	1.558200272	0.98791466		4.388987456	3.057376124
	1.693300026	1.012310015		4.769523345	3.132874321
	1.589499949	1.044341432		4.477149353	3.232004431
	1.721600235	1.13865125		4.849236632	3.52387234

Supplementary Table 8. KYSE30 MTS raw results (knockdown)

	raw			normalized	
day0	NC	si-fam175b	day0	NC	si-fam175b
	0.278942678	0.229641368		1.017604677	0.994465652
	0.297478924	0.245789316		1.085226351	1.064394602
	0.254578327	0.23367892		0.928721622	1.011950336
	0.265467822	0.21456782		0.96844735	0.929189409
AVERAGE	0.274116938	0.230919356			
day1	NC	si-fam175b	day1	NC	si-fam175b
	0.37799876	0.400597051		1.378969	1.734792
	0.432875326	0.06096271		1.579163	0.264
	0.451521308	0.06096271		1.647185	0.264
	0.446328711	0.06096271		1.628242	0.264
day2	NC	si-fam175b	day2	NC	si-fam175b
	0.691325932	0.723832424		2.522011	3.134568
	0.700767068	0.74977529		2.556453	3.246914
	0.691915832	0.802619799		2.524163	3.475758
	0.691325932	0.779595061		2.522011	3.376049
day3	NC	si-fam175b	day3	NC	si-fam175b
	0.995698527	1.236468314		3.632386	5.354546
	0.931690577	1.148894919		3.39888	4.975308
	1.023903789	1.136455063		3.735281	4.921437
	1.006942256	1.220192425		3.673404	5.284063

Supplementary Table 9. EC9706 MTS raw results (knockdown)

	raw			normalized	
day0	NC	si-fam175b	day0	NC	si-fam175b
	0.235946742	0.278545722		0.937909934	1.054972007
	0.289635679	0.279812457		1.151328382	1.059769675
	0.22273678	0.244924689		0.885399124	0.927634748
	0.257946895	0.252842679		1.02536256	0.957623569
AVERAGE	0.251566524	0.264031387			
day1	NC	si-fam175b	day1	NC	si-fam175b
	0.366710371	0.444503664		1.457707349	1.683525847
	0.404316125	0.484325952		1.60719367	1.834349916
	0.427923255	0.474518616		1.701034178	1.797205332
	0.413698007	0.505896336		1.644487513	1.916046203
day2	NC	si-fam175b	day2	NC	si-fam175b
	0.639546581	0.743348232		2.542256302	2.815378282
	0.625252667	0.755792664		2.485436682	2.862510678
	0.631208276	0.757214832		2.509110774	2.867897038
	0.574682308	0.741570456		2.284414868	2.808645082
day3	NC	si-fam175b	day3	NC	si-fam175b
	0.906675689	1.246992648		3.604119	4.722895499
	0.875488853	1.305659256		3.480148466	4.945091082
	0.884289964	1.302696384		3.51513369	4.933869416
	0.917574267	1.341185208		3.647441848	5.079643085

Supplementary Table 10. P53 mutation in TCGA database

V173G	V216E	V274G	W91*	C124*	C135Lfs*14
0	0	0	0	1	1
C135Y	C176Y	C229Lfs*18,C229F	C238*	C238_N239delinsY	C242Y
1	1	1	1	1	1
C275F	C275F,G245V	D42Ifs*2	E198*	E204*	E224*
1	1	1	1	1	1
E285V,X187_splice	E294*	E294*,T125=	E294Sfs*51	E343*	F109S
1	1	1	1	1	1
F134L	G154Afs*16,S94*	H168R	H179R	H193R	I195F
1	1	1	1	1	1
I195N,R209K	I255del	L130V	L252Sfs*93	P151S	P250_I251delinsL
1	1	1	1	1	1
P278H	P278S	P64Qfs*84	Q317*	R110L	R158H,X126_splice
1	1	1	1	1	1
R158L	R175G,C275Vfs*70	R175G,R110C	R213*	R213*,C135R	R248Q,D391Tfs*31,P390H
1	1	1	1	1	1
R249W,R249M,I251F	R273C,V197L	R273H,V272M	R282G	R282W	R306*
1	1	1	1	1	1
R306*,K164Sfs*6	S106R	V122Dfs*26	V157F	V272M,R174W	V274L
1	1	1	1	1	1
W146*	X126_splice	X224_splice	X225_splice	X261_splice	X307_splice
1	1	1	1	1	1
X332_splice	Y126S	Y163C	Y205C	Y220C	Y220C,R282W
1	1	1	1	1	1
Y220C,R342*	Y220D	P128Lfs*42	P278A	R248Q	R273C
1	1	2	2	2	2
R273H	X187_splice	R175H	NaN		
2	3	4	9		
